# Adding Water to the Mill: Olmesartan-Induced Collagenous Sprue—A Case Report and Brief Literature Review

**DOI:** 10.1155/2016/4837270

**Published:** 2016-04-21

**Authors:** Claudine Desruisseaux, Michaël Bensoussan, Etienne Désilets, Hanh-Khiem Tran, Robert Arcand, Germain Poirier, Andrew Wisniewski, Thibaut Manière

**Affiliations:** ^1^Gastroenterology Service, Charles-LeMoyne Hospital, Sherbrooke University, Greenfield Park, QC, Canada J4V 2H1; ^2^Pathology Department, Charles-LeMoyne Hospital, Sherbrooke University, Greenfield Park, QC, Canada J4V 2H1; ^3^Intensive Care Unit, Charles-LeMoyne Hospital, Sherbrooke University, Greenfield Park, QC, Canada J4V 2H1

## Abstract

Collagenous sprue (CS) is a distinct clinicopathological disorder histologically defined by a thickened subepithelial band (Freeman, 2011). It is a rare condition which has been recently observed in a significant proportion of sprue-like enteropathy associated with olmesartan, a novel entity described by Rubio-Tapia et al. in 2012. CS is historically associated with a poor prognosis (Marthey et al., 2014). However, histological and clinical improvements have been described in most studies with concomitant usage of corticosteroids and/or gluten-free diet (Marthey et al., 2014). We report a unique case of olmesartan-induced collagenous sprue in a 79-year-old man that showed complete histological and clinical remission with the sole withdrawal of the incriminating drug. The literature on this topic is briefly reviewed.

## 1. Case Presentation

A 79-year-old man was referred to the emergency room for a three-week history of severe diarrhea. His past medical history was remarkable for chronic kidney disease with a baseline creatinine level of 170 *μ*mol/L and hypertension. His medication was amlodipine 5 mg and olmesartan 40 mg daily started 3 years ago.

The patient had been experiencing worsening watery diarrhea, which had lately increased to as many as 10 episodes a day. He also reported diffuse abdominal pain, nausea, emesis, and a weight loss of 10 kg in 3 weeks.

On admission, the vital signs showed significant hypotension responding to volemic repletion. Antihypertensive medication was withheld. The physical examination showed mucosal dehydration and generalized abdomen tenderness without guarding. Laboratory investigations showed an acute renal failure associated with a metabolic acidosis (creatinine: 782 *μ*mol/L; urea: 33 *μ*mol/L; and bicarbonate: 9.9 mmol/L). The blood cells count, electrolytes, and albumin level were unremarkable.

**Figure 1:**
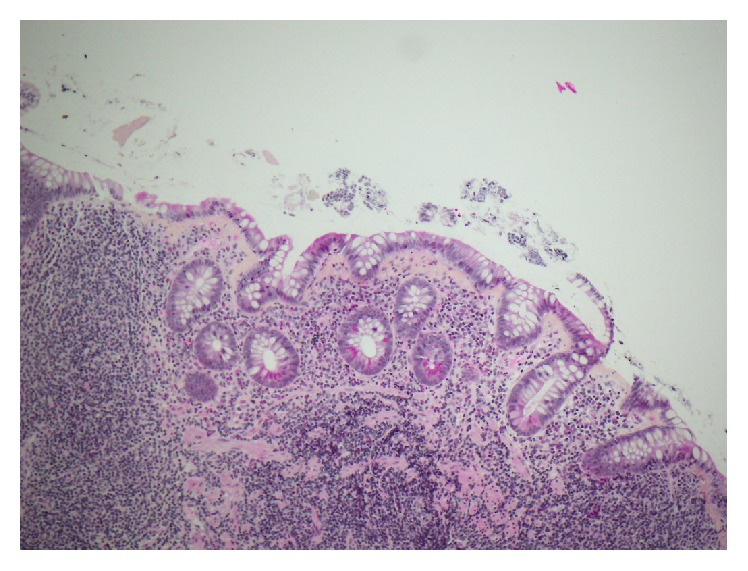
The ileal biopsies of the patient showed pathognomonic findings of collagenous sprue including villous atrophy with thickened collagen table (HPS, 10x).

The patient was admitted to the ICU and an alkaline fluid resuscitation with bicarbonate was able to prevent dialysis. The upper GI endoscopy was unremarkable except for a Mallory-Weiss tear and the ileocolonoscopy was normal. Biopsies were taken from the duodenum, terminal ileum, and colon. The duodenal and ileal histologic findings showed a collagenous sprue (CS) characterized by complete villous atrophy with increased intraepithelial lymphocytes and thickened collagen table (Figure 1). Colonic pathologic findings were negative. Stool analyses for infectious agents and tissue IgA transglutaminase antibodies were negative.

**Figure 2:**
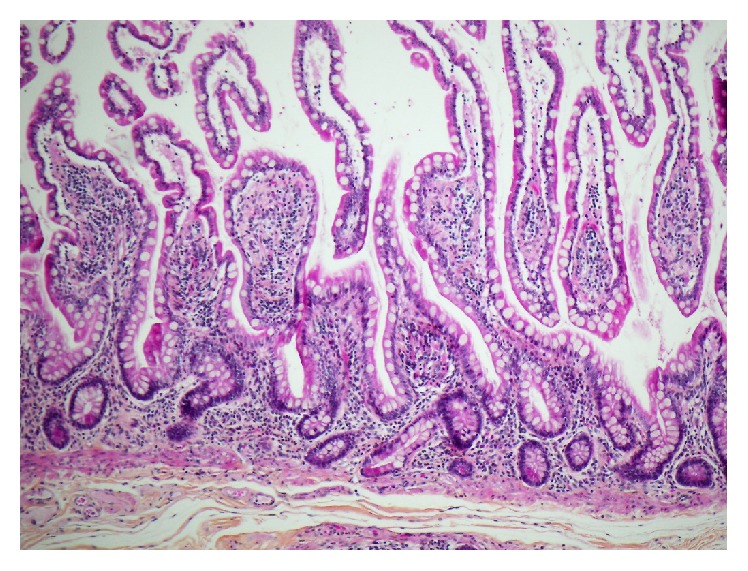
Normal ileal mucosa 4 months following cessation of olmesartan (HPS, 10x).

Given the histopathologic findings, olmesartan-induced enteropathy was suspected and the drug was withheld indefinitely. Within days, the patient noticed a slight improvement of the diarrhea. Two months later, the symptoms had subsided and repeat endoscopy with biopsies demonstrated a complete histologic remission of the CS (Figure 2).

## 2. Discussion

CS is a rare form of enteropathy that has been classically regarded as a complication of refractory celiac disease, an association that remains controversial [1]. In the past decade, a number of new causes of CS have been recognized: notably, the drug-induced form of enteropathy. In 2010, Rubio-Tapia et al. conducted one of the largest case series on CS to date and found that a significant proportion of the study cohort were taking olmesartan, an angiotensin II receptor antagonist commonly used for the management of hypertension [2]. Interestingly, the same group of authors later introduced sprue-like enteropathy associated with olmesartan, therefore broadening the differential diagnosis of seronegative villous atrophy enteropathy [3]. A warning related to the risk of sprue-like enteropathy has since been implemented for all olmesartan single and combination products.

Historically, it was often felt that the prognosis of patients presenting with CS was grim. However, complete histological resolution of olmesartan-induced CS has been demonstrated after the cessation of olmesartan but more often with concomitant use of corticosteroid and/or gluten-free diet, which may reveal itself to be a confusion factor pertaining to the clinical evolution [2, 3]. Very few series have documented both clinical and histological resolution with the sole withdrawal of olmesartan. In a recent national French study reporting 36 cases of olmesartan-induced enteropathy, complete recovery without being exposed to systemic steroids and/or immunosuppressants has been documented in only four cases [4].

Indeed, since the recognition of olmesartan-associated enteropathy by Rubio-Tapia et al., our patient is the first reported case of olmesartan-induced CS in Canada that achieved complete clinical and histological resolution with sole withdrawal of the incriminating drug. This leads us to believe that this condition is likely to be underreported and that patients are exposed to unnecessary high dose corticosteroids treatments in attempt to treat this condition. Furthermore, considering the potential reversibility, we encourage physicians to be aware of this clinicopathological entity and include the latter in the differential diagnosis of seronegative villous atrophy with collagenous band thickening.
